# Smokers’ support for the ban on sale of slim cigarettes in six European countries: findings from the EUREST-PLUS ITC Europe Surveys

**DOI:** 10.12688/openreseurope.13405.2

**Published:** 2022-07-05

**Authors:** Enkeleint A. Mechili, Krzysztof Przewoźniak, Pete Driezen, Christina N Kyriakos, Charis Girvalaki, Ute Mons, Anne CK Quah, Esteve Fernández, Antigona C Trofor, Tibor Demjén, Paraskevi A Katsaounou, Witold Zatoński, Geoffrey T Fong, Constantine I Vardavas

**Affiliations:** 1 Department of Health Care, Faculty of Health,, University of Vlora, Vlora, 9401, Albania; 2 European Network for Smoking and Tobacco Prevention, Chausse d'lxelles 144, Brussels, 1050, Belgium; 3 School of Medicine, University of Crete, Voutes, Heraklion, 71409, Greece; 4 Maria Skłodowska-Curie National Research Institute of Oncology, 15B Wawelska Street, Warsaw, 02-034, Poland; 5 Health Promotion Foundation, Mszczonowska, Nadarzyn, 05-830, Poland; 6 Collegium Civitas, plac Defilad 1, Warsaw, 00-901, Poland; 7 Department of Psychology, University of Waterloo, Waterloo, Ontario, N2L 3G1, Canada; 8 School of Public Health Sciences, University of Waterloo, University Avenue West 200, Waterloo, Ontario, N2L 3G1, Canada; 9 Department of Primary Care and Public Health, School of Public Health, Imperial College London, 310 Reynolds Building, St. Dunstan's Road, London, W6 9RP, UK; 10 Cancer Prevention Unit and WHO Collaborating Centre for Tobacco Control, German Cancer Research Center (DKFZ), Im Neuenheimer Feld 280, Heidelberg, 69120, Germany; 11 Heart Center, Faculty of Medicine and University Hospital Cologne, University of Cologne, Kerpener Straße 62, Cologne, 50937, Germany; 12 Tobacco Control Research Group, Bellvitge Biomedical Research Institute (IDIBELL), Av Gran Via De L’Hospitalet 199-203, L’Hospitalet de Llobregat, Catalonia, 08908, Spain; 13 School of Medicine and Health Sciences, Bellvitge Campus, Universitat de Barcelona, L’Hospitalet de Llobregat, Catalonia, 08908, Spain; 14 Center for Biomedical Research in Respiratory Diseases (CIBER of Respiratory Diseases, CIBERES), Instituto de Salud Carlos III, C/ Monforte de Lemos 3-5, Madrid, 28029, Spain; 15 Tobacco Control Unit, Catalan Institute of Oncology (ICO), Av Gran Via De L’Hospitalet 199-203, L’Hospitalet de Llobregat, Catalonia, 08908, Spain; 16 University of Medicine and Pharmacy ‘Grigore T. Popa’ Iasi, Strada Universității 16, Iași, 700115, Romania; 17 AerPur Romania, Street Argentina 35 Sector 1, Bucharest, 011753, Romania; 18 Smoking or Health Hungarian Foundation, Fiumei 18/B IB IV LPH I 2, Budapest, 1044, Hungary; 19 First ICU Evaggelismos Hospital Athens, National and Kapodistrian University of Athens, Ipsilantou 45-47, Athens, 10676, Greece; 20 European Observatory of Health Inequalities, President Stanisław Wojciechowski State University of Applied Sciences, Nowy Świat 4 st, Kalisz, 62-800, Poland; 21 Ontario Institute for Cancer Research, 661 University Avenue, Toronto, Ontario, M5G 0A3, Canada

**Keywords:** Tobacco Products Directive, slim cigarettes, tobacco policy, European Union

## Abstract

**Background:** Efforts to regulate tobacco products and reduce consumption in the European Union (EU) include the European Tobacco Products Directive (TPD), which went into force in May 2016. Despite the initial discussion to include a ban on sale of slim cigarettes, it was excluded in the final TPD. The main goal of this study was to examine support for a ban on slim cigarettes among smokers in six European Countries.

**Methods:** Data from the 2018 (Wave 2) International Tobacco Control Policy Evaluation Project 6 European Country (ITC 6E) EUREST-PLUS project survey, a cross sectional study of adult smokers (n=5592) from Germany, Greece, Hungary, Poland, Romania, and Spain, was analysed. Descriptive statistics were used to estimate support for a ban on slim cigarettes by sociodemographic characteristics and smoking behaviors. Logistic regression analysis was used to examine factors associated with support for a ban on slim cigarettes and perceptions of harm.

**Results:** Support for a ban on slims varied across countries, with highest support in Romania (33.8%), and lowest in Greece (18.0%). Female smokers (OR=0.78; 95%CI=0.67-0.91, daily smokers (OR=0.68; 95%CI=0.47-0.97), menthol smokers (OR=0.55; 95%CI=0.36-0.86), and smokers who did not have plans to quit within next six months (OR=0.45; 95%CI=0.36-0.56) had significantly lower odds of supporting a ban on slim cigarettes. Overall, 21% of smokers perceived slim cigarettes as less harmful than regular cigarettes.

**Conclusions:** Support for a ban of slim cigarettes was relatively low among smokers, while misperceptions that slim cigarettes are less harmful is high, particularly among countries where slim cigarette use is more prevalent. Findings support a ban on slim cigarettes to reduce misperceptions around slim cigarettes being less harmful.

## Introduction

Around 700,000 people die annually in the European Union (EU) due to tobacco use ^1^. Despite recent efforts undertaken to reduce misperceptions of tobacco products, implementation of tobacco control policies is still needed ^2^.

Within the EU countries, around half of smokers use cigarettes with special characteristics such as additive-free or organic, light, menthol flavor, slim, etc. Slim cigarettes are consumed by around 5% of EU smokers ^1^. Slim cigarettes have a diameter of 5 or 6 millimeters and a length that varies from 68–121 mm according to the category (from regular to extra-long) ^3^. Tobacco companies have historically targeted women by promoting slim cigarettes as stylish and feminine ^3^. A qualitative study among young women concluded that slim cigarettes are perceived to be correlated with glamour, cleanliness, and safety ^4^. Similarly, in a Scottish study, slim cigarettes were considered as lovely, pretty, fun, cool, and cute by teenage girls and young women ^5^. A Polish study among young population aged 13–19 years (secondary and high school students) found that that slim cigarettes were perceived to be less harmful ^6^. In light of such misperceptions, supporters (both smokers and non-smokers) of a ban on slim cigarettes argued that slim cigarettes are as harmful as normal cigarettes but that their promotion and design features reinforce smoker’s misconceptions on these products to be less harmful and, therefore, make quitting smoking more difficult. A study conducted by Wang *et al.* (2021) ^7^ concluded that to date no evidence suggests that slim cigarettes are less hazardous than traditional products ^7^. Similarly, also the National Cancer Institute states that any kind of cigarettes increases the risk of smoking-related cancers and other diseases ^8^.

The revised EU Tobacco Products Directive (TPD) came into force in May 2016. The TPD aims to improve protection of the health of EU citizens from the harms of tobacco ^9^. The main objective of the TPD provisions was to reduce misperceptions around reduced harm often associated with special characteristics of tobacco products. Such features of cigarettes and roll-your-own tobacco banned under the TPD include characterizing flavors, misleading labels, packs resembling a food or cosmetic product and pack sizes less than 20 cigarettes or 30g of tobacco, but did not include information on banning slim cigarettes. Population support for tobacco control policies is an important determinant of successful policy implementation. However, there is a lack of data on the factors that are associated with harm perceptions and support for a slim cigarettes ban in the EU. Given that the TPD implementation report is being prepared in 2021, it is important to understand support for banning slim cigarettes. The main goal of this study was to examine support for a ban on slim cigarettes among a representative sample of smokers in six EU countries.

## Methods

The survey protocols and all materials, including the survey questionnaires, information letter and consent, were cleared for ethics by the ethics research committee at the University of Waterloo (Ontario, Canada, REB#21262), and ethics committees in Germany (Ethikkommission der Medizinischen Fakultät Heidelberg, No. DV-097-160401), Greece (Medical School, University of Athens - Research and Ethics Committee, No. 156023993), Hungary (Medical Research Council – Scientific and Research Committee, No. 46344-2/2017/EKY), Poland (State College of Higher Vocational Education - Committee and Dean of the Department of Health Care and Life Sciences, No. dnia 22.09.2017 r), Romania (Iuliu Hatieganu University of Medicine and Pharmacy, No. 155), and Spain (Clinical Research Ethics Committee of Bellvitge, Hospital Universitari de Bellvitge, Catalonia, PR100/16). Eligible respondents were male or female adult (aged 18+) cigarette smokers who reported smoking at least once a month. After a respondent was selected, an information letter was provided and consent was taken. Informed written consent for participation and publication of data was obtained at the time of data collection, in which participants were informed that, “All personal information you provide is treated as strictly confidential, subject to legal requirements and limitations. It will be held in secure storage and password protected at the University of Waterloo, Canada and only be accessed by this research team. Any identifying information about you will be removed before the data are securely stored, so that your answers cannot be linked back to you. After two years, the survey data, but not your name or other identifying information, will be shared with authorized researchers in other countries, as it will be used to make comparisons of smoking behaviour and attitudes across countries.”

Data came from the 2018 (Wave 2) International Tobacco Control Evaluation Project 6 European Country (ITC 6E) Survey, a cohort study of 6,027 adult smokers (aged 18+ years) who reported having smoked at least 100 cigarettes in their lifetime and smoked at least monthly from Germany, Greece, Hungary, Poland, Romania, and Spain. These respondents consisted of two sample types: (1) re-contacted (cohort) respondents (n=3,195) from Wave 1 of the ITC 6E Survey who were followed up regardless of their current smoking status (retention rates ranged from 36% in Hungary to 71% in Germany and Spain, with an average of 53% for the full sample). Participants were recruited via multi-stage stratified random sampling. Sampling was based on geographic strata created according to Nomenclature of Territorial Units for Statistics (NUTS) regions and degree of urbanisation; and (2) new respondents (current smokers) to replenish those who were lost to attrition. The replenishment sample (n=2,832) was recruited from newly selected households, and approached in the same manner as Wave 1, with the random-walk procedure beginning at a new (random) starting point. Smokers followed from Wave 1 who had quit smoking by Wave 2 were excluded from this analysis (n=415).

Face-to-face interviews were conducted from February to May 2018. After providing written consent, respondents completed the survey via a computer-assisted personal interview conducted in each country's official language. The ITC 6E Survey is part of the European Commission Horizon-2020 funded study European Regulatory Science on Tobacco: Policy Implementation to Reduce Lung Diseases (EUREST_PLUS-HCO-06-2015), which aimed to evaluate the impact of the EU TPD ^10^. Further details about the study methodology are provided elsewhere ^11– 14^.

The primary outcome of this study, support for a ban on slim cigarettes, was assessed with the question: “Would you support or oppose a law that banned all slim cigarettes, that is, those cigarettes that are slimmer in size than regular cigarettes?” (strongly support, support, oppose, strongly oppose, don’t know). Demographic measures were sex (male, female), age group (18–24, 25–39, 40–54, 55+), degree of urbanisation (low, intermediate, high) ^11, 12^, education (low, moderate, high), and income (low, moderate, high, not reported). In all countries, low education was defined as pre-primary/no education, primary, and lower secondary. Moderate education was defined as upper secondary, post-secondary non-tertiary, and short-cycle tertiary. High education was defined as bachelor or equivalent, master or equivalent, and doctoral or equivalent. Country-specific income thresholds were used to classify household income into low, moderate, or high ^15^. Respondents who refused to provide income information were classified as income not reported. Additional independent measures were smoking behaviours, as defined in Table 1. Smoking status was classified as daily or non-daily (at least once a week or at least once a month). Unless otherwise indicated, respondents who provided “refused” or “don’t know” responses for any of the measures were excluded from the analysis.

**Table 1.** Support for a ban on slim cigarettes among smokers from six European countries, International Tobacco Control Evaluation Project Six European Country Survey Wave 2 (2018) (N=5577).

	% Supporting Slim Ban *			Odds of Support ^†^		Test		
Covariate	(Unwtd Freq)	%	(95% CI)	aOR	(95% CI)	ChiSq	DF	p
**Country**								
Germany	(182 / 939)	19.2	(14.6, 24.9)	0.99	(0.64, 1.52)	23.95	5	<.001
Greece	(174 / 944)	18.0	(13.7, 23.3)	0.91	(0.61, 1.37)			
Hungary	(188 / 942)	21.7	(16.7, 27.8)	1.12	(0.77, 1.64)			
Poland	(177 / 947)	20.0	(15.5, 25.4)	1.00	(0.68, 1.47)			
Romania	(291 / 916)	33.8	(29.2, 38.8)	2.00	(1.41, 2.82)			
Spain	(193 / 889)	20.2	(16.9, 24.1)	1.00				
**Urban/rural residence**								
Urban	(389 / 1932)	21.8	(18.5, 25.5)	1.14	(0.82, 1.57)	1.19	2	0.550
Intermediate	(487 / 2134)	22.7	(20.0, 25.8)	1.18	(0.88, 1.57)			
Rural	(329 / 1511)	21.8	(18.1, 26.1)	1.00				
**Sex**								
Female	(536 / 2715)	19.5	(17.4, 21.9)	0.78	(0.67, 0.91)	10.03	1	0.002
Male	(669 / 2862)	24.2	(21.9, 26.6)	1.00				
**Age group**								
18–24	(96 / 432)	24.1	(19.1, 30.0)	1.22	(0.88, 1.71)	1.94	3	0.586
25–39	(351 / 1533)	22.9	(20.2, 25.8)	1.12	(0.90, 1.39)			
40–54	(416 / 1940)	21.7	(19.2, 24.4)	1.10	(0.89, 1.35)			
55+	(342 / 1672)	21.1	(18.3, 24.1)	1.00				
**Income**								
Not stated	(260 / 1543)	17.7	(14.9, 20.9)	0.88	(0.65, 1.19)	12.23	3	0.007
Low	(226 / 993)	22.3	(18.7, 26.3)	1.14	(0.84, 1.54)			
Moderate	(452 / 1800)	25.8	(22.8, 29.1)	1.34	(1.05, 1.71)			
High	(267 / 1241)	22.4	(18.9, 26.3)	1.00				
**Education**								
Low	(379 / 1790)	21.5	(18.6, 24.8)	1.08	(0.79, 1.49)	0.47	2	0.790
Moderate	(698 / 3126)	22.8	(20.5, 25.3)	1.10	(0.83, 1.46)			
High	(121 / 640)	19.9	(16.3, 24.1)	1.00				
**Smoking status**								
Daily smoker	(1135 / 5343)	21.8	(19.8, 23.9)	0.68	(0.47, 0.97)	4.46	1	0.035
Non-daily smoker	(70 / 234)	29.3	(23.4, 35.9)	1.00				
**Cigarettes smoked/day**								
≤ 10	(485 / 2072)	23.1	(20.7, 25.7)	0.98	(0.62, 1.54)	1.60	3	0.659
11–20	(577 / 2796)	21.4	(19.1, 23.9)	0.92	(0.61, 1.41)			
21–30	(91 / 475)	22.2	(17.4, 27.9)	1.11	(0.69, 1.80)			
31+	(46 / 214)	21.7	(14.8, 29.9)	1.00				
**Flavour smoked**								
Menthol	(44 / 277)	15.2	(10.8, 20.5)	0.55	(0.36, 0.86)	8.44	3	0.038
Other flavour	(20 / 115)	19.8	(11.0, 31.6)	0.79	(0.40, 1.59)			
Unflavoured	(974 / 4545)	22.0	(20.0, 24.2)	0.95	(0.73, 1.23)			
No usual brand	(167 / 640)	25.9	(21.5, 30.9)	1.00				
**Smokes factory-made(FM)/roll-your-own(RYO)**								
FM	(919 / 4165)	22.7	(20.6, 24.8)	1.00	(0.73, 1.37)	0.24	2	0.885
RYO	(204 / 1027)	20.5	(16.9, 24.6)	1.06	(0.73, 1.52)			
Both	(79 / 382)	20.2	(15.6, 25.9)	1.00				
**Attempts to quit in past year**								
Has not tried to quit in last 12 months	(989 / 4773)	21.2	(19.2, 23.3)	0.91	(0.72, 1.15)	0.57	1	0.452
Tried to quit last 12 months	(215 / 798)	27.8	(23.9, 32.1)	1.00				
**Plans to quit smoking**								
No plans to quit	(983 / 4966)	20.1	(18.2, 22.2)	0.45	(0.36, 0.56)	48.35	1	<.001
Plans to quit in next 6 months	(222 / 611)	38.8	(34.1, 43.7)	1.00				
**Harm of own cigarette brand vs. other brands**								
Don't know	(14 / 129)	12.7	(6.1, 22.4)	0.58	(0.29, 1.19)	2.99	3	0.394
A little less harmful	(191 / 803)	25.2	(21.6, 29.3)	1.04	(0.82, 1.30)			
A little more harmful	(66 / 199)	31.8	(24.9, 39.8)	1.17	(0.80, 1.72)			
No different	(932 / 4444)	21.4	(19.3, 23.7)	1.00				
**Slim cigarettes are less harmful than regular cigarettes**								
Don't know	(80 / 532)	16.5	(12.9, 20.9)	0.79	(0.58, 1.08)	50.06	3	<.001
Strongly agree/Agree	(402 / 1190)	33.4	(29.3, 37.6)	2.14	(1.69, 2.71)			
Neither	(264 / 1249)	22.2	(18.8, 25.9)	1.26	(0.99, 1.60)			
Strongly disagree/disagree	(457 / 2589)	18.3	(16.0, 20.9)	1.00				

* Weighted percent † Odds ratios from a weighted multivariable logistic regression model including all covariates listed in table. Unwtd freq – unweighted frequency; CI – confidence interval; aOR, adjusted odds ratio; ChiSq, chi-square statistic; DF – degrees of freedom. 65 respondents were excluded from the logistic regression model due to missing data (refused/don’t know responses) for one or more independent variables.

Descriptive statistics were used to estimate support for a ban on slim cigarettes by sociodemographic characteristics and smoking behaviors. Wald χ ^2^ tests were used to test for an association between independent variables and support for a ban on slim cigarettes. Multivariable logistic regression was used to estimate adjusted odds ratios (OR) and the corresponding 95% confidence intervals (CI). The ‘support for a slim ban’ variable was transformed into a binary variable (supports or strongly supports vs. otherwise = oppose/strongly oppose/don’t know). Additionally, respondents stated whether they believe that slim cigarettes are less harmful than regular cigarettes (agree/strongly agree that slims are less harmful vs. neither agree nor disagree/disagree/strongly disagree/don’t know). All statistical analyses were conducted using SAS-callable SUDAAN (Version 11.0.3) to account for the complex sampling design and cross-sectional sampling weights. 

## Results

### Sample characteristics

Of 5592 respondents who smoked on a daily (96%) or non-daily basis (4%), 51% were male, 28% were 25–39 years of age, 35% were 40–54 years of age, and 30% were 55 or older. About 35% of respondents from Germany, Hungary, Poland, and Romania lived in urban areas, 53% of respondents from Spain lived in urban areas, while only 17% of respondents from Greece lived in urban areas. Across all countries, more than half of all respondents (56%) had a moderate education and 12% had a high education; 32% had a moderate household income, 22% had a high income, and 28% chose not to report their income.

### Support for a ban on slim cigarettes

Support for a ban on slims varied across the six European countries, with highest support in Romania (33.8%; 95%CI=29.2-38.8), and lowest in Greece (18.0%; 95%CI=13.7-23.3). Smokers from Romana had significantly higher odds (aOR=2.00; 95%CI =1.41-2.82) of supporting a ban on slim cigarettes than smokers from Spain. Those who had a moderate income in comparison to those with high income (aOR=1.34; 95%CI =1.05-1.71) were more likely to support a ban on slim cigarettes. Female smokers (aOR=0.78; 95%CI=0.67-0.91; ref=males), daily smokers (aOR=0.68; 95%CI=0.47-0.97; ref=non-daily smokers), menthol smokers (aOR=0.55; 95%CI=0.36-0.86; ref=no usual brand), and smokers who did not have plans to quit within next six months (aOR=0.45; 95%CI=0.36-0.56; ref=smokers with plans to quit in the next six months) had significantly lower odds of supporting a ban on slim cigarettes. Smokers who agreed or strongly agreed that slim cigarettes are less harmful than regular cigarettes were more likely to support a ban on slim cigarettes compared to those who disagreed or strongly disagreed (OR=2.14, 95%CI=1.69-2.71) ( Table 1).

### Perceptions that slim cigarettes are less harmful

Perceptions that slim cigarettes are less harmful than regular cigarettes also varied significantly across the six countries, ranging from 8.1% (95%CI=5.8-11.3) among German smokers to over one-quarter among Hungarian (29.1%; 95%CI=24.0-34.8), Polish (28.9%, 95%CI=24.3-33.9), and Romanian (26.8%, 95%CI=23.0-31.1) smokers. Smokers who reported not having plans to quit within the next six months were less likely to report that slim cigarettes are less harmful compared to those with plans to quit (aOR=0.70; 95%CI=0.54-0.91). Lastly, smokers who believed that their own cigarette brand is less harmful than other brands were more likely to report that slims are less harmful compared to those who perceived no difference in harm between their own and others’ brand (aOR=1.84; 95%CI=1.45-2.33) ( Table 2).

**Table 2.** Perceptions that slim cigarettes are less harmful among smokers from six European countries, International Tobacco Control Evaluation Project Six European Country Survey Wave 2 (2018) (N=5592).

	% Slims Less Harmful			Odds Slims Less Harmful ^†^		Test		
Covariate	(Unwtd Freq)	% *	(95%CI)	aOR	(95%CI)	ChiSq	DF	p
**Country**								
Germany	(75 / 939)	8.1	(5.8, 11.3)	0.34	(0.21, 0.53)	71.32	5	<.001
Greece	(132 / 951)	13.1	(10.0, 17.0)	0.63	(0.42, 0.94)			
Hungary	(263 / 949)	29.1	(24.0, 34.8)	1.77	(1.22, 2.56)			
Poland	(290 / 947)	28.9	(24.3, 33.9)	1.48	(1.04, 2.11)			
Romania	(252 / 918)	26.8	(23.0, 31.1)	1.23	(0.86, 1.78)			
Spain	(180 / 888)	19.5	(15.7, 24.0)	1.00				
**Urban/rural residence**								
Urban	(405 / 1943)	19.6	(17.1, 22.3)	0.80	(0.60, 1.06)	2.78	2	0.249
Intermediate	(433 / 2137)	20.1	(17.6, 23.0)	0.92	(0.69, 1.24)			
Rural	(354 / 1512)	24.0	(20.3, 28.1)	1.00				
**Sex**								
Female	(624 / 2724)	22.3	(20.3, 24.5)	1.17	(1.00, 1.37)	4.02	1	0.045
Male	(568 / 2868)	19.8	(17.8, 22.0)	1.00				
**Age group**								
18–24	(95 / 432)	20.5	(16.2, 25.6)	0.83	(0.60, 1.15)	2.67	3	0.445
25–39	(324 / 1538)	20.7	(18.2, 23.4)	0.87	(0.71, 1.08)			
40–54	(390 / 1948)	20.1	(17.7, 22.7)	0.86	(0.70, 1.06)			
55+	(383 / 1674)	22.4	(19.9, 25.2)	1.00				
**Income**								
Not stated	(353 / 1555)	21.5	(18.5, 24.8)	0.91	(0.69, 1.18)	2.14	3	0.544
Low	(189 / 995)	18.8	(15.6, 22.4)	1.00	(0.73, 1.36)			
Moderate	(389 / 1801)	21.1	(18.5, 23.9)	1.08	(0.86, 1.36)			
High	(261 / 1241)	21.6	(18.6, 25.0)	1.00				
**Education**								
Low	(358 / 1799)	20.1	(17.3, 23.2)	1.03	(0.75, 1.42)	0.69	2	0.708
Moderate	(703 / 3129)	21.8	(19.9, 23.9)	1.10	(0.82, 1.46)			
High	(125 / 645)	18.8	(15.1, 23.1)	1.00				
**Smoking status**								
Daily smoker	(1153 / 5356)	21.1	(19.4, 23.0)	1.38	(0.86, 2.21)	1.76	1	0.185
Non-daily smoker	(39 / 236)	16.4	(11.2, 22.8)	1.00				
**Cigarettes smoked/day**								
≤ 10	(456 / 2085)	21.9	(19.7, 24.4)	1.14	(0.71, 1.82)	5.88	3	0.118
11–20	(614 / 2795)	21.8	(19.5, 24.2)	1.14	(0.72, 1.79)			
21–30	(79 / 477)	13.0	(10.0, 16.8)	0.77	(0.47, 1.26)			
31+	(34 / 215)	17.0	(11.6, 23.8)	1.00				
**Flavour smoked**								
Menthol	(84 / 278)	26.5	(20.4, 33.7)	0.79	(0.52, 1.18)	7.17	3	0.067
Other flavour	(33 / 114)	28.0	(15.2, 44.1)	1.41	(0.70, 2.82)			
Unflavoured	(900 / 4562)	19.7	(17.9, 21.6)	0.75	(0.58, 0.98)			
No usual brand	(175 / 638)	25.8	(21.5, 30.7)	1.00				
**Smokes factory-made/roll-your-own**								
FM	(942 / 4175)	21.8	(20.1, 23.7)	1.25	(0.85, 1.83)	7.86	2	0.020
RYO	(180 / 1032)	18.2	(14.8, 22.2)	0.90	(0.58, 1.41)			
Both	(68 / 382)	18.1	(13.5, 23.9)	1.00				
**Attempts to quit in past year**								
Has not tried to quit in last 12 months	(1013 / 4782)	21.0	(19.2, 23.0)	1.13	(0.89, 1.44)	1.03	1	0.310
Tried to quit last 12 months	(178 / 804)	20.6	(17.6, 23.9)	1.00				
**Plans to quit smoking**								
No plans to quit	(1012 / 4974)	20.0	(18.2, 21.9)	0.70	(0.54, 0.91)	7.23	1	0.007
Plans to quit in next 6 months	(180 / 615)	28.8	(24.5, 33.5)	1.00				
**Harm of own cigarette brand vs. other brands**								
Don't know	(30 / 133)	21.8	(13.8, 31.9)	0.86	(0.50, 1.48)	27.57	3	<.001
A little less harmful	(240 / 804)	30.6	(26.3, 35.2)	1.84	(1.45, 2.33)			
A little more harmful	(63 / 199)	28.9	(22.0, 36.9)	1.37	(0.92, 2.04)			
No different	(858 / 4454)	18.9	(17.1, 20.8)	1.00				

* Weighted percent. † Odds ratios from a weighted multivariable logistic regression model including all covariates listed in table. Unwtd freq – unweighted frequency; CI – confidence interval; aOR, adjusted odds ratio; ChiSq, chi-square statistic; DF – degrees of freedom. 51 respondents were excluded from the logistic regression model due to missing data (refused/don’t know responses) for one or more independent variables

## Discussion

This study has found that approximately 20% of smokers supported a ban on slim cigarettes. Country, gender, daily/non-daily smoking status, and quitting plans were key correlates of supporting a ban on slim cigarettes. Women were less likely to support a ban on slims than men, which is not surprising given that slim cigarettes have historically been specifically marketed to females by the tobacco industry ^16^. The fact that slim cigarettes are associated with perceptions of glamour, cleanliness, and safety ^4^, which are mainly characteristics of femininity, may be the reason ^5, 17^. According to the 2017 Special Eurobarometer 458, women in the EU are more likely than men to smoke slim cigarettes (10% vs 2%) and find slim cigarettes attractive (e.g., feminine, elegant) (20% vs. 15%) ^1^. Non-daily smokers and those smokers planning to quit were more likely to support a ban of slim cigarettes. This is similar to a study that found that menthol smokers who had plans to quit were more likely to support a menthol ban in comparison to those with no plans to quit ^18^.

We found considerable country differences in reporting that slim cigarettes are less harmful, with misperceptions significantly higher in Hungary, Poland, and Romania (>25%) than in Germany (8%). Variation may be partly explained by differences in popularity of slim cigarettes. According to the 2017 Eurobarometer, use of slim cigarettes among the observed countries also varied (1% in Spain, 2% in Germany, 5% in Hungary, 6% in Greece, 6% in Romania, and 16% in Poland). Given that slim cigarettes are as harmful as other cigarettes, the high levels of misperceptions that slim cigarettes are less harmful in our sample of EU smokers is concerning. However, we found some counter-intuitive results that smokers who agreed (rather than disagreed) that slim cigarettes are less harmful were more likely to a support a ban on slim cigarettes. While the existing literature is limited, other studies have found harm perceptions and social norms to be associated with support for tobacco control policies and related to the choice of tobacco product used ^19– 21^.

While the initial draft TPD included a ban on slim cigarettes, the final version lacked such a ban. This was proposed to be due to the strong tobacco industry lobbying to dilute the regulation ^22^. As previous research has shown that smokers perceptions are impacted by cigarette pack and stick design, and as other provisions included in the TPD were aimed at reducing misperceptions about reduced harm, results from this study further illustrate how banning slim cigarettes would support this goal ^23, 24^.

While this study uses data from large representative samples of smokers from six EU Member States, there are some limitations to be considered. First, the survey did not directly ask respondents if they smoke slim cigarettes, which precluded analysis of this variable as a possible covariate. Second, the cross-sectional nature of the analysis does not allow for any causal conclusions. Lastly, our study relies on self-report data, which might be subject to information bias, leading to misclassification.

In conclusion, our results indicate that among a sample of smokers from six EU Member States, the percentage of smokers who support a slims ban is low, while misperceptions that slim cigarettes are less harmful is high, particularly among countries where slim cigarette use is more prevalent. Findings of the current study provide evidence that can be used when discussing a ban on slim cigarette in order to further mitigate misperceptions that certain tobacco products are less harmful than others. The European Commission should consider this evidence in the next revision of the TPD. 

## Funding

The ITC EUREST-PLUS Project has received funding from the European Union’s Horizon 2020 research and innovation programme under grant agreement No 681109 (CIV) and the University of Waterloo (GTF). Additional support was provided to the University of Waterloo by the Canadian Institutes of Health Research (FDN-148477). GTF was supported by a Senior Investigator Grant from the Ontario Institute for Cancer Research. EF is partly supported by the Ministry of Universities and Research, Government of Catalonia (2017SGR319). UM is supported by the Marga and Walter Boll Foundation (Kerpen, Germany).

## Data availability

In each country participating in the international Tobacco Control Policy Evaluation (ITC) Project, the data are jointly owned by the lead researcher(s) in that country and the ITC Project at the University of Waterloo. Data from the ITC Project are available to approved researchers 2 years after the date of issuance of cleaned data sets by the ITC Data Management Centre. Researchers interested in using ITC data are required to apply for approval by submitting an International Tobacco Control Data Repository (ITCDR) request application and subsequently to sign an ITCDR Data Usage Agreement. The criteria for data usage approval and the contents of the Data Usage Agreement are described online ( http://www.itcproject.org). 
