# Supplementary material for: Smokers’ support for the ban on sale of slim cigarettes in six European countries: findings from the EUREST-PLUS ITC Europe Surveys
Source: Open Res Eur. 2023 Jun 19;1:52. Originally published 2021 May 18. [Version 4] doi: 10.12688/openreseurope.13405.4 (PMC10446023; doi:10.12688/openreseurope.13405.4)
Supplement: Peer review history

# Peer review: Reviewer response for version 4

**DOI:** 10.21956/openreseurope.17478.r32951

**Published:** 2023-07-04

**Authors:** Jaana Sepp

**Affiliations:** 1 Academic and International Affairs Office, Tallinn Health Care College, Tallinn, Estonia

The authors have made commendable improvements to the article based on the feedback provided by the reviewers. The inclusion of potential biases that could impact the study results is a crucial addition, as it enhances the transparency and strengthens the interpretation of the findings. By explicitly addressing these biases, the authors have shown a commitment to providing a comprehensive and rigorous analysis.

 The authors have also taken into account the complexity of the statistical analyses used, particularly in relation to the complex sampling design and weighting adjustments. By discussing the assumptions, limitations, and potential pitfalls associated with these analyses, the authors have demonstrated a clear understanding of the statistical nuances and have provided readers with a better understanding of the strengths and limitations of the study.

 The expanded discussion on the implications of the findings and future research directions is a notable improvement. By exploring strategies to address misperceptions about the harm of slim cigarettes and considering the effectiveness of different tobacco control policies, the authors have shown a proactive approach towards addressing a significant public health issue. This addition enhances the practical relevance of the study and provides valuable insights for policymakers and researchers.

 Furthermore, the authors' acknowledgment of the complexity of tobacco consumption and the need for multifaceted approaches is a commendable addition to the article. By recognizing that banning slim cigarettes alone will not solve the problem, the authors highlight the importance of comprehensive tobacco control strategies that encompass various activities and actions. This nuanced perspective adds depth to the discussion and reflects a well-rounded understanding of the topic.

 Overall, the authors' improvements demonstrate a high level of responsiveness to the reviewers' feedback and a commitment to enhancing the scientific soundness of the article. The revised version reflects a comprehensive analysis, thoughtful considerations of biases and limitations, and valuable insights for future research and policy development.

Is the study design appropriate and does the work have academic merit?

Yes

Is the work clearly and accurately presented and does it cite the current literature?

Yes

If applicable, is the statistical analysis and its interpretation appropriate?

Yes

Are all the source data underlying the results available to ensure full reproducibility?

Yes

Are the conclusions drawn adequately supported by the results?

Yes

Are sufficient details of methods and analysis provided to allow replication by others?

Yes

Reviewer Expertise:

Safety management, occupational health and safety, human behaviour.

I confirm that I have read this submission and believe that I have an appropriate level of expertise to confirm that it is of an acceptable scientific standard.

# Peer review: Reviewer response for version 3

**DOI:** 10.21956/openreseurope.16334.r31297

**Published:** 2023-05-19

**Authors:** Camilla Wikström-Grotell

**Affiliations:** 1 Mälardalen University, Västerås, Sweden; 2 Arcada UAS, Helsinki, Finland

Your paper, which deals with an important problem regarding public health, is on an overall level well-structured and written. Objective, method and results are well reported. I only miss a clarification of the problem with smoking and the banning of slim cigarettes in a deeper understanding of the problem from a health promotion perspective, which includes preventive and health behaviour change interventions.

 The main goal of this study was to examine support for a ban on slim cigarettes among smokers in six European Countries. You aim to answer a specific question, which you do. However, the smoking is a multicomplex problem that is based on nicotine dependence but also on social and psychological factors. It can be seen as a chronic, severe disease, which should be prevented and which is difficult to treat. The problem cannot be solved simply by banning certain tobacco products. It is important to demonstrate this understanding of the problem in the introduction, and possibly follow this up in the discussion with some type of statement that the ban is a step in the right direction but not sufficient as only measure or intervention. This can be supported with one or a few relevant sources.

Is the study design appropriate and does the work have academic merit?

Yes

Is the work clearly and accurately presented and does it cite the current literature?

Yes

If applicable, is the statistical analysis and its interpretation appropriate?

Yes

Are all the source data underlying the results available to ensure full reproducibility?

Yes

Are the conclusions drawn adequately supported by the results?

Partly

Are sufficient details of methods and analysis provided to allow replication by others?

Yes

Reviewer Expertise:

My areas of expertise are health promotion, health behavior and evidence-based health care & medicine, qualitative research methods. I am not an expert on statistical methods.

I confirm that I have read this submission and believe that I have an appropriate level of expertise to confirm that it is of an acceptable scientific standard, however I have significant reservations, as outlined above.

# Response

**Published:** 2023-06-08

**Authors:** Enkeleint A. Mechili

**Affiliations:** University of Vlora, Albania

Point by point. We would like to warmly thank the reviewers for their kind words and valuable suggestions which improve significantly this paper. We have followed them strictly in order to improve our manuscript. All our revisions are with track changes in the revised manuscript. **Review 1:**   Summary: The article investigates the support for a ban on slim cigarettes among smokers in six European countries and explores the factors associated with this support. The study employs descriptive statistics, Wald χ2 tests, and multivariable logistic regression to analyze data obtained from a large representative sample of smokers. The findings indicate that approximately 20% of smokers supported a ban on slim cigarettes, with significant variations across countries. Gender, smoking status, quitting plans, and perceptions of harm were identified as key correlates of supporting a ban. The article highlights the importance of addressing misperceptions regarding the harm of slim cigarettes and recommends the inclusion of a ban on slim cigarettes in tobacco control policies.

 Response 1: Thank you very much for your general comment. **Review 2:** Strengths:  

1. Utilization of a representative sample: The study benefits from a large representative sample of smokers from six European countries, which enhances the generalizability of the findings to the target population.
2. Use of statistical analyses: The study employs appropriate statistical methods, including descriptive statistics, Wald χ2 tests, and multivariable logistic regression, to examine the relationships between independent variables and support for a ban on slim cigarettes.
3. Inclusion of relevant covariates: The article includes covariates in the regression models that have been previously used in constructing sampling weights and identified in empirical research on slim cigarettes and perceptions of tobacco product harm, which strengthens the validity of the analysis.

Response 2: Thank you for pointing some of the key strengths of the current article. It is true that the number of participants, the statistical analyses used, and the inclusion of covariates are some key strengths of the current article.

**Review 3:** Weaknesses and Suggestions for Improvement:

1. Limited discussion on potential sources of bias: The article fails to discuss potential biases that could impact the findings, such as social desirability bias or recall bias. Addressing these potential biases explicitly will allow readers to better interpret the results and understand their limitations.
2. Limited discussion of statistical limitations: The article does not adequately discuss the assumptions, limitations, and potential pitfalls associated with the statistical analyses employed, particularly in the context of the complex sampling design and weighting adjustments. Providing a clear discussion of these statistical limitations will help readers understand the strengths and limitations of the study's findings.
3. Incomplete implications and future research discussion: The article could benefit from an expanded discussion of the implications of the findings and potential future research directions. Specifically, exploring strategies to address misperceptions about the harm of slim cigarettes and assessing the effectiveness of different tobacco control policies would enhance the practical relevance and impact of the study.

  Response 3: We would like toto warmly thank the reviewer for the kind and important comments that improve significantly the current article. We have responded to the raised issues accordingly. Please see below for each comment our response as well as in the text the proposed changes. **Review 4:** Discuss potential biases, such as social desirability bias or recall bias, that may have influenced the self-report data and provide strategies to mitigate their impact on the study findings.

   Response 4: We warmly thank the reviewer for this very important comment. We have added a sentence about this limitation in the limitation paragraph. In this paragraph we mentioned that *“Moreover, due to the nature of the study some possible bias can be such as* *social desirability bias and/or societal impact”.* We consider that is better not to include recall bias in this case as the data collected are not of a retrospective nature. However, if the reviewer consider important to include, we will do it with great pleasure. **Review 5:** Provide a thorough discussion of the assumptions, limitations, and potential pitfalls associated with the statistical analyses used, particularly considering the complex sampling design and weighting adjustments.   Response 5: Thank you for the comment. We have added the below text and reference. The statistical approach used for analysis of the ITC 6 European Country (ITC 6E) Survey data is considered standard for the analysis of population health survey data employing complex sampling designs using unequal weighting and clustering. Statistical approaches that ignore the complex design will produce variances and probability levels of hypothesis tests that are too small, potentially leading to erroneous conclusions (LaVange et al., 1996). Therefore, it was essential to account for the sampling design and sampling weights in the analysis of the ITC 6E data. The statistical procedures implemented in SUDAAN provide the ability to correctly compute variances and hypothesis tests under complex sampling when only minimal design information is employed (LaVange et al., 1996). However, the approximations used are generally considered to be conservative, so that estimated variances will be slightly larger (LaVange et al., 1996); thus, p-values reported here may be somewhat conservative. Reference to add: LaVange LM, Stearns SC, Lafata JE, Koch GG, Shah BV. Innovative strategies using SUDAAN for analysis of health surveys with complex samples. *Statistical Methods in Medical Research,* 1996; 5: 311-329. https://doi.org/10.1177/096228029600500306 **Review 6:** Expand the implications and future research discussion to explore strategies for addressing misperceptions about the harm of slim cigarettes and assessing the effectiveness of different tobacco control policies. This would enhance the practical relevance and impact of the study.   Response 6: Thank you for your important comment. In the end of the manuscript, we have added two sentences about this issue. *“Future activities should focus on the* *strategies for addressing misperceptions about the harm of slim cigarette. Moreover, it is important policy makers to assess the effectiveness of different tobacco control policies and to better implement them in different EU settings”.* **Review 7:** Your paper, which deals with an important problem regarding public health, is on an overall level well-structured and written. Objective, method and results are well reported. I only miss a clarification of the problem with smoking and the banning of slim cigarettes in a deeper understanding of the problem from a health promotion perspective, which includes preventive and health behaviour change interventions.   Response 7: Thank you for your comment. The banning of the slim cigarettes can improve health of the population as they have similar ingredients to normal cigarettes. We have mentioned some of the key health problems at references 7 and 8. Moreover, we added a short text about the impact of the slim cigarettes on health status. We have added a reference that strengthen this issue. *“From all the above it is clear that the slim cigarette consumption could improve the health status of the population. Exposure to some toxicants such as formaldehyde, ammonia and the phenols increases in super slim cigarettes according to a study conducted in Canada ^9^.” * Moreover, at the end of the manuscript we added a text that focuses on prevention and health promotion campaigns. **Review 8:**

 The main goal of this study was to examine support for a ban on slim cigarettes among smokers in six European Countries. You aim to answer a specific question, which you do. However, the smoking is a multicomplex problem that is based on nicotine dependence but also on social and psychological factors. It can be seen as a chronic, severe disease, which should be prevented and which is difficult to treat. The problem cannot be solved simply by banning certain tobacco products. It is important to demonstrate this understanding of the problem in the introduction, and possibly follow this up in the discussion with some type of statement that the ban is a step in the right direction but not sufficient as only measure or intervention. This can be supported with one or a few relevant sources.   Response 8: Thank you for the very important and significant comment. We have added a text and a reference to strengthen this issue. *“Tobacco is a multicomplex problem that is not depending just on the nicotine dependence but also on social and psychological factors. A study in San Francisco shows that banning of flavored tobacco use can decrease the prevalence but not in high rates ^10^. This clearly states that banning is not the only measure possible but comprehensive polices are needed for tobacco control.”*

# Peer review: Reviewer response for version 3

**DOI:** 10.21956/openreseurope.16334.r31298

**Published:** 2023-05-16

**Authors:** Jaana Sepp

**Affiliations:** 1 Academic and International Affairs Office, Tallinn Health Care College, Tallinn, Estonia

Summary: The article investigates the support for a ban on slim cigarettes among smokers in six European countries and explores the factors associated with this support. The study employs descriptive statistics, Wald χ2 tests, and multivariable logistic regression to analyze data obtained from a large representative sample of smokers. The findings indicate that approximately 20% of smokers supported a ban on slim cigarettes, with significant variations across countries. Gender, smoking status, quitting plans, and perceptions of harm were identified as key correlates of supporting a ban. The article highlights the importance of addressing misperceptions regarding the harm of slim cigarettes and recommends the inclusion of a ban on slim cigarettes in tobacco control policies.

Strengths:

1. Utilization of a representative sample: The study benefits from a large representative sample of smokers from six European countries, which enhances the generalizability of the findings to the target population.
2. Use of statistical analyses: The study employs appropriate statistical methods, including descriptive statistics, Wald χ2 tests, and multivariable logistic regression, to examine the relationships between independent variables and support for a ban on slim cigarettes.
3. Inclusion of relevant covariates: The article includes covariates in the regression models that have been previously used in constructing sampling weights and identified in empirical research on slim cigarettes and perceptions of tobacco product harm, which strengthens the validity of the analysis.

Weaknesses and Suggestions for Improvement:

1. Limited discussion on potential sources of bias: The article fails to discuss potential biases that could impact the findings, such as social desirability bias or recall bias. Addressing these potential biases explicitly will allow readers to better interpret the results and understand their limitations.
2. Limited discussion of statistical limitations: The article does not adequately discuss the assumptions, limitations, and potential pitfalls associated with the statistical analyses employed, particularly in the context of the complex sampling design and weighting adjustments. Providing a clear discussion of these statistical limitations will help readers understand the strengths and limitations of the study's findings.
3. Incomplete implications and future research discussion: The article could benefit from an expanded discussion of the implications of the findings and potential future research directions. Specifically, exploring strategies to address misperceptions about the harm of slim cigarettes and assessing the effectiveness of different tobacco control policies would enhance the practical relevance and impact of the study.

Scientifically Sound Recommendations:

1. Discuss potential biases, such as social desirability bias or recall bias, that may have influenced the self-report data and provide strategies to mitigate their impact on the study findings.
2. Provide a thorough discussion of the assumptions, limitations, and potential pitfalls associated with the statistical analyses used, particularly considering the complex sampling design and weighting adjustments.
3. Expand the implications and future research discussion to explore strategies for addressing misperceptions about the harm of slim cigarettes and assessing the effectiveness of different tobacco control policies. This would enhance the practical relevance and impact of the study.

By addressing these points, the authors can strengthen the scientific soundness of the article, improve its transparency, and provide a more comprehensive understanding of the factors associated with support for a ban on slim cigarettes among smokers in the European context.

Is the study design appropriate and does the work have academic merit?

Yes

Is the work clearly and accurately presented and does it cite the current literature?

Yes

If applicable, is the statistical analysis and its interpretation appropriate?

Yes

Are all the source data underlying the results available to ensure full reproducibility?

Yes

Are the conclusions drawn adequately supported by the results?

Yes

Are sufficient details of methods and analysis provided to allow replication by others?

Yes

Reviewer Expertise:

Safety management, occupational health and safety, human behaviour.

I confirm that I have read this submission and believe that I have an appropriate level of expertise to confirm that it is of an acceptable scientific standard, however I have significant reservations, as outlined above.

# Peer review: Reviewer response for version 2

**DOI:** 10.21956/openreseurope.16103.r29650

**Published:** 2022-08-31

**Authors:** Anish Thekkumkara Surendran Nair

**Affiliations:** 1 Community Medicine, Government Medical College Thiruvananthapuram, Thiruvananthapuram, Kerala, India

I found that the authors addressed all my previous queries. I hereby approve the manuscript.

Is the study design appropriate and does the work have academic merit?

Yes

Is the work clearly and accurately presented and does it cite the current literature?

Yes

If applicable, is the statistical analysis and its interpretation appropriate?

Partly

Are all the source data underlying the results available to ensure full reproducibility?

Yes

Are the conclusions drawn adequately supported by the results?

Yes

Are sufficient details of methods and analysis provided to allow replication by others?

Yes

Reviewer Expertise:

NA

I confirm that I have read this submission and believe that I have an appropriate level of expertise to confirm that it is of an acceptable scientific standard.

# Peer review: Reviewer response for version 2

**DOI:** 10.21956/openreseurope.16103.r29649

**Published:** 2022-08-15

**Authors:** Shu Xu

**Affiliations:** 1 New York University, New York, NY, USA

The aim of this manuscript was to identify the factors that are associated with perceptions and support for a slim cigarette ban in the EU. This review emphasized the statistical methodology and results reporting. The manuscript failed to adequately address major issues below.

1. In Introduction, authors added two sentences to justify that “slim cigarettes are less harmful than cigarettes” is a misperception. Two citations were added. One was Wang *et al* (2021), titled “Slim cigarette smoking in Urban China: Who are the early adopters and why,” which provided no empirical evidence and did not conclude on “slim cigarettes are less harmful.” The other citation from the NCI website referred to no specific published articles. Authors were supposed to conduct a literature search more thoroughly.
2. Study population and participants. It was unclear why data from established cigarettes smokers were used to study the perceptions and support for a slim cigarette ban. What is the rationale behind it? It was also unclear what the study population was.
3. Study variables:
  Authors have added more descriptions about the measures in the revised version. Some of the measures were presented in the paragraph for data analysis plan, which is inappropriate.
  Importantly, authors need to explain why potential covariates were selected for this study based on literature review. For example, it was unclear why “Harm of own cigarette brand vs other brands” reported by cigarette smokers was considered to study “Odds of support for slim cigarette ban.
  Results could be more interpretable if reference category of a categorical variable were properly selected. For example, it is difficult to interpret an odds ratio when using “No usual brand" as the reference for Flavor Smoked. Typically, the ‘Unflavored” is used as the reference.

Is the study design appropriate and does the work have academic merit?

Yes

Is the work clearly and accurately presented and does it cite the current literature?

Yes

If applicable, is the statistical analysis and its interpretation appropriate?

Partly

Are all the source data underlying the results available to ensure full reproducibility?

Partly

Are the conclusions drawn adequately supported by the results?

Partly

Are sufficient details of methods and analysis provided to allow replication by others?

No

Reviewer Expertise:

NA

I confirm that I have read this submission and believe that I have an appropriate level of expertise to state that I do not consider it to be of an acceptable scientific standard, for reasons outlined above.

# Response

**Published:** 2022-09-09

**Authors:** Enkeleint A. Mechili

**Affiliations:** University of Vlora, Albania

We would like to warmly thank the reviewers for the kind words and valuable suggestions which improve significantly this paper. We have followed them strictly in order to improve our manuscript. All our revisions are with track changes in the revised manuscript.  

***Reviewer Comment 1:*** *The aim of this manuscript was to identify the factors that are associated with perceptions and support for a slim cigarette ban in the EU. This review emphasized the statistical methodology and results reporting. The manuscript failed to adequately address major issues below. In Introduction, authors added two sentences to justify that “slim cigarettes are less harmful than cigarettes” is a misperception. Two citations were added. One was Wang et al (2021), titled “Slim cigarette smoking in Urban China: Who are the early adopters and why,” which provided no empirical evidence and did not conclude on “slim cigarettes are less harmful.” The other citation from the NCI website referred to no specific published articles. Authors were supposed to conduct a literature search more thoroughly.*

**Response 1**: Thank you very much for your kind comment. We have replaced these articles with some more appropriate according to your comments. The text added now is: The study of Siu et al. concluded that is a misconception to consider less harmful the super slim cigarette. ^7 ^Authors report that this kind of tobacco can emission higher levels of toxicants such as phenol, ammonia etc. while the amount of nicotine is similar to other regular cigarettes ^7^.  Another study found that levels of cadmium were found 20% and 27% higher on women smoking long and ultra-long cigarette in comparison to normal cigaretts ^8^.  

***Reviewer Comment 2:*** *Study population and participants. It was unclear why data from established cigarettes smokers were used to study the perceptions and support for a slim cigarette ban. What is the rationale behind it? It was also unclear what the study population was.*

**Response 2:**

- The analysis was based on current smokers only because smokers (and not quitters) are directly affected by regulations banning the sale of slim cigarettes. This is consistent with other studies using ITC data examining support for different tobacco control policies (for example, Smith et al., 2021).
- The study population is described in more detail in the second paragraph of the methods section, describing the total number of respondents participating in Wave 2 of the ITC EUREST-PLUS Survey, the number of smokers followed from Wave 1 who quit smoking (n=415), and the number of current smokers remaining who were eligible for analysis (n=5612).

***Reviewer Comment 3:*** *Study variables: Authors have added more descriptions about the measures in the revised version. Some of the measures were presented in the paragraph for data analysis plan, which is inappropriate. Importantly, authors need to explain why potential covariates were selected for this study based on literature review. For example, it was unclear why “Harm of own cigarette brand vs other brands” reported by cigarette smokers was considered to study “Odds of support for slim cigarette ban. Results could be more interpretable if reference category of a categorical variable were properly selected. For example, it is difficult to interpret an odds ratio when using “No usual brand" as the reference for Flavor Smoked. Typically, the ‘Unflavored” is used as the reference.*

**Response 3:**

- The statistical analysis paragraph was revised so that the description of “support for a slim ban” and “beliefs about the harm of slim cigarettes” was moved to the previous paragraph describing all measures used for analysis. This ensures that the statistical analysis paragraph only describes the statistical methods used.
- Standard sociodemographic measures were included in regression models as well as variables used to construct the sampling weights (see Thompson *et al.* 2020 and ITC Project, 2018, reference #s 11 and 14 in the paper). Other covariates were included in regression models based on previous empirical research on slim cigarettes (e.g., Mutti *et al*., 2011; Minaker *et al*., 2017) or perceptions of the harmfulness of different tobacco products (e.g., Gravely et al., 2020). This additional information was added to the paragraph describing the measures. The “harm of own cigarette brand vs other brands” was included as a covariate in the model examining support for a slim ban because respondents were not directly asked whether they smoked a slim brand. However, previous research (e.g., Mutti *et al*., 2011; Ford *et al*., 2016) found that people who smoked slim cigarettes were more likely to believe their own brand was less harmful than other brands. Therefore, perceptions about the harm of one’s own brand might be related to support for a ban on slim cigarettes and was included as a covariate.
- The menthol variable was recoded so that “unflavoured” formed the reference category in regression models. Tables 1 and 2 were revised to reflect this change.

 Additional references to add:

- Mutti S, Hammond D, Borland R, Cummings KM, O’Connor RJ, Fong GT. Beyond light and mild: cigarette brand descriptors and perceptions of risk in the International Tobacco Control (ITC) Four Country Survey. *Addiction.* 2011; **106**: 1166-1175.
- Minaker LM, Tait H, Ong M, Nguyen N. Slim cigarette smoking prevalence among Canadian youth smokers: Implications for federal standardized packaging legislation. *Canadian Journal of Public Health.* 2017; **108(5-6)**: e565-e570.
- Ford A, Moodie C, Purves R, MacKintosh AM. Adolescent girls and young adult women’s perceptions of superslims cigarette packaging: a qualitative study. *BMJ Open.* 2016; **6**:e010102.
- Gravely S, Driezen P, Kyriakos CN, Thompson ME, Balmford J, Demjén, Fernández E, Mons U, Tountas Y, Janik-Koncewicz K, Zatoński W, Trofor AC, Vardavas CI, Fong GT. European adult smokers’ perceptions of the harmfulness of e-cigarettes relative to combustible cigarettes: cohort findings from the 2016 and 2018 EUREST-PLUS ITC Europe Surveys. *European Journal of Public Health.* 2020; **30 (Suppl 3)**: iii38-iii45.
- Smith TT, Nahhas GJ, Borland R. Cho YJ, Chung-Hall J, Fairman RT, Fong GT, McNeill A, Popova L, Thrasher JF, Cummings KM. Which tobacco control policies do smokers support? Findings from the International Tobacco Control Four Country Smoking and Vaping Survey. *Preventive Medicine.* 2021; **149**: 106600.

# Peer review: Reviewer response for version 1

**DOI:** 10.21956/openreseurope.14475.r28904

**Published:** 2022-04-19

**Authors:** Shu Xu

**Affiliations:** 1 New York University, New York, NY, USA

The aim of this manuscript was to identify the factors that are associated with perceptions and support for a slim cigarettes ban in the EU. This review emphasized the statistical methodology and results reporting.

1. Introduction: Add a few sentences to explain why “slim cigarettes are less harmful” than cigarettes is a misperception. Explain why this is misspecification. In the same paragraph, explain what are the correct perceptions.
2. Methods.

- To allow replication by other researchers, authors need to provide a complete and detailed introduction to all the study variables and their measures. Specifically, authors need to provide (1) a complete set of dependent and independent variables, (2) all response categories of categorical, and (3) all the response categories of a categorical variable if it was recoded. For example, it is unclear how one of the dependent variables, “support of a slim ban,” was recoded. The authors stated that the response to the original variable has three categories (i.e., “support,” “oppose,” and “don’t know.”). In another paragraph, the authors stated the response categories as “supports or strong supports, ” “otherwise.” It is also unclear how these responses were recoded into binary responses (i.e., “oppose/strong oppose/don’t know”). A similar problem exists in the other dependent variable. Another example is the variable, “income.” It is unclear how the “Low,” “Moderate,” and “High” income categories were defined.  This problem exists in multiple other independent variables.
                                                        
- One of the dependent variables, “perceptions that slim cigarettes are less harmful” was recoded into two response categories (agree/strongly agree that slims are less harmful v.s. neither agree nor disagree/disagree/strong disagree/don’t know). Those who responded “neither agree nor disagree” may be considered as “slim cigarette smokers are not less or more harmful than cigarettes.” However, those who “disagree/strongly disagree” considered “slim cigarette smokers are more harmful than cigarettes.” This mixture of different responses in the reference group may lead to biased results, hence, invalid conclusions. Authors need to justify why the reference group contains multiple responses (“neither agree nor disagree/disagree/strongly disagree/don’t know”) with citations, and discuss its implication. Alternatively, authors may consider the dependent variable multi-categorical.
-  If R was not used for data analysis, then remove the following sentence. “The analyses  can be done in most software packages that handle complex data design, such as R, an open-access software, using the  ‘svytable” and “svyglm” commands from the survey package.”
- Add 1-2 sentences to explain the size of the study sample, and how the authors handled missing data.

3. Results.

- Add one paragraph summarizing the characteristics of the study sample.
- Replace “OR” with “aOR” in text and Tables 1 & 2 because multivariable logistic regressions were conducted. Update the footnotes accordingly.

Is the study design appropriate and does the work have academic merit?

Yes

Is the work clearly and accurately presented and does it cite the current literature?

Yes

If applicable, is the statistical analysis and its interpretation appropriate?

Partly

Are all the source data underlying the results available to ensure full reproducibility?

Partly

Are the conclusions drawn adequately supported by the results?

Partly

Are sufficient details of methods and analysis provided to allow replication by others?

No

Reviewer Expertise:

My area is biostatistics. This review emphasized the statistical methodology and results reporting.

I confirm that I have read this submission and believe that I have an appropriate level of expertise to state that I do not consider it to be of an acceptable scientific standard, for reasons outlined above.

# Peer review: Reviewer response for version 1

**DOI:** 10.21956/openreseurope.14475.r28929

**Published:** 2022-04-05

**Authors:** Anish Thekkumkara Surendran Nair

**Affiliations:** 1 Community Medicine, Government Medical College Thiruvananthapuram, Thiruvananthapuram, Kerala, India

It is a well written article based on ITC6E data.

 However, the following points may be addressed

1. Under 'Methods' section, it is written that "Once a respondent was selected, an information letter was provided and consent was taken". But the selection criteria was not clearly mentioned.
2. It is given that "The primary outcome of this study, support for a ban on slim cigarettes, was assessed with the question: “Would you support or oppose a law that banned all slim cigarettes, that is, those cigarettes that are slimmer in size than regular cigarettes?” (support; oppose; don’t know)". But it is not clear that how this variable was used in analysis. Is it support vs non-support, counting 'don't know' category also along with 'oppose' group.
3. Last part of 'Methods' session, it is given that "The analyses can be done in most software packages that handle complex data design, such as R, an open-access software, using the ‘svytable” and “svyglm” commands from the survey package." It is not clear whether the investigators used the statistical package. If not, this sentence could be deleted.

Is the study design appropriate and does the work have academic merit?

Yes

Is the work clearly and accurately presented and does it cite the current literature?

Yes

If applicable, is the statistical analysis and its interpretation appropriate?

Partly

Are all the source data underlying the results available to ensure full reproducibility?

Yes

Are the conclusions drawn adequately supported by the results?

Yes

Are sufficient details of methods and analysis provided to allow replication by others?

Yes

Reviewer Expertise:

Public Health and Epidemiology

I confirm that I have read this submission and believe that I have an appropriate level of expertise to confirm that it is of an acceptable scientific standard, however I have significant reservations, as outlined above.
